# Persistent platelet activation and apoptosis in virologically suppressed HIV-infected individuals

**DOI:** 10.1038/s41598-018-33403-0

**Published:** 2018-10-09

**Authors:** Emersom C. Mesquita, Eugenio D. Hottz, Rodrigo T. Amancio, Alan B. Carneiro, Lohanna Palhinha, Lara E. Coelho, Beatriz Grinsztejn, Guy A. Zimmerman, Matthew T. Rondina, Andrew S. Weyrich, Patrícia T. Bozza, Fernando A. Bozza

**Affiliations:** 1 Laboratório de Medicina Intensiva, Instituto Nacional de Infectologia Evandro Chagas (INI), Fundação Oswaldo Cruz (FIOCRUZ), Rio de Janeiro, Brazil; 2 Laboratório de HIV, Instituto Nacional de Infectologia Evandro Chagas (INI), Fundação Oswaldo Cruz (FIOCRUZ), Rio de Janeiro, Brazil; 3 Laboratório de Imunofarmacologia, Instituto Oswaldo Cruz (IOC) – Fundação Oswaldo Cruz (FIOCRUZ), Rio de Janeiro, Brazil; 4 Departamento de Bioquímica, Instituto de Ciências Biológicas – Universidade Federal de Juiz de Fora (UFJF), Juiz de Fora, Minas Gerais Brazil; 5 D’Or Institute for Research and Education (IDOR), Rio de Janeiro, Brazil; 6 Molecular Medicine Program and Department of Internal Medicine, University of Utah, Salt Lake City, Utah USA

## Abstract

Cardiovascular diseases and thrombotic events became major clinical problems in the combined antiretroviral therapy (cART) era. Although the precise mechanisms behind these clinical problems have not been fully elucidated, a persistent pro-inflammatory state plays a central role. As platelets play important roles on both, thrombus formation and inflammatory/immune response, we aimed at investigating platelet function in HIV-infected subjects virologically controlled through cART. We evaluate parameters of activation, mitochondrial function and activation of apoptosis pathways in platelets from 30 HIV-infected individuals under stable cART and 36 healthy volunteers. Despite viral control achieved through cART, HIV-infected individuals exhibited increased platelet activation as indicated by P-selectin expression and platelet spreading when adhered on fibrinogen-coated surfaces. Platelets from HIV-infected subjects also exhibited mitochondrial dysfunction and activation of apoptosis pathways. Finally, thrombin stimuli induced lower levels of P-selectin translocation and RANTES secretion, but not TXA_2_ synthesis, in platelets from HIV-infected individuals compared to control; and labeling of platelet alpha granules showed reduced granule content in platelets from HIV-infected individuals when compared to healthy subjects. In summary, platelets derived from HIV-infected individuals under stable cART exhibit a phenotype of increased activation, activation of the intrinsic pathway of apoptosis and undermined granule secretion in response to thrombin.

## Introduction

Combined antiretroviral therapy (cART) is the cornerstone in the treatment of human immunodeficiency virus 1 (HIV-1)-infected individuals^1,2^. Despite the viral control achieved through cART, HIV-infected individuals experience higher rates of long-term comorbidities such as cardiovascular diseases and non-AIDS cancers^3–7^. Nowadays, ischemic thrombotic events represent one of the most frequent long-term complications and cause of death among virologically suppressed HIV-1 individuals^7–11^. Many of long-term complications in HIV-infected individuals, including cardiovascular events, are related to continuing immune suppression^3,9,10^ and associated with a persistent inflammatory state^12–14^.

Platelets are highly specialized and essential effector cells in hemostasis that present major roles in pathological thrombosis. Aside from their role in coagulation, platelets are also major inflammatory cells with key roles in innate and adaptive immune responses^15,16^. Activated platelets mediate inflammatory and immune responses by a variety of mechanisms, including release of stored chemokines and interactions with leukocytes through P-selectin-mediated adhesion^16–18^. It has been previously shown that HIV-infected individuals present increased platelet activation^19–23^. Platelet activation correlates with measures of immune and inflammatory activation in HIV-infected individuals^19–21^, and the levels of P-selectin surface expression associate with viral loads^19,20,22^. However, it is not known whether platelet activation persists in HIV-infected individuals virologically suppressed through ART.

Leukocytes are highly susceptible to apoptosis in HIV-1-infected individuals^24–26^. Intrinsic cell death pathways, originally described to nucleated cells, were recently demonstrated in platelets^27,28^, but whether platelet apoptosis takes place in HIV-infected individuals has not been previously investigated. Here we show that platelets from subjects living with HIV under stable ART present increased platelet activation compared to healthy volunteers. We also demonstrate increased mitochondrial dysfunction and activation of the intrinsic pathway of apoptosis in platelets from HIV-infected individuals. We found evidence that chronic activation in platelet from HIV-infected subjects lead to platelet exhaustion of granule stored factors and lower granule translocation and secretion after thrombin stimulation. Our data indicate that, despite viral suppression, HIV-infected individuals under stable ART present sustained platelet activation and dysfunction, which may impact on persistent inflammation and long-term comorbidities as cardiovascular diseases.

## Results

### Clinical characteristics of healthy volunteers and HIV-infected individuals

The demographic and clinical characteristics of the study populations are summarized in Table 1. All HIV-infected individuals were under stable cART and had undetectable viral load at study inclusion time. Two nucleoside reverse-transcriptase inhibitors (NRTIs) plus one non-nucleoside reverse-transcriptase inhibitor (NNRTI) was the most prevalent combination of antiretrovirals (56%). The median duration of ART up to inclusion was 63.5 months (IQR 38–82). The median CD4 cell count at inclusion was 569 cells/mm^3^ (IQR 242–866), and the median time elapsed since HIV-1 diagnosis was 114 months (IQR 63–121). There were no differences in platelet counts and coagulation parameters between HIV-infected individuals and healthy volunteers (Table 1).

**Table 1:** Clinical and laboratory characteristics of controls and HIV-infected individuals.

	Control (n = 36)	HIV + (n = 30)
Age (years)	30 (27–35)	45 (38–50)*
Gender (male)	14 (38.8%)	14 (46.6%)
TCD4^+^ cell count (per mL)	—	526 (258–862)
Nadir TCD4^+^	—	243 (71–550)
HIV-1 Viral load (x 10^3^ copies/mL)	—	Undetectable
Time since HIV diagnosis (months)	—	114 (63–121)
cARV	—	30 (100%)
NRTI + NNRTI	—	18 (60%)
NRTI + PI/R	—	9 (30%)
NRTI + INSTI	—	3 (10%)
Platelet count (x 10^3^)	196 (153–238)	205.8 (143–213)
APT (%)	71 (67–87)	85 (70–94)
PTT (Seconds)	30 (28–32)	29 (24–31)
INR	1,18 (1,0–1,2)	1,1 (1,0–1,2)

Results are expressed as median and interquartile range (IQR). NRTI, nucleoside reverse transcriptase inhibitor; NNRTI, non-nucleoside reverse transcriptase inhibitor; PI, protease inhibitor; INSTI, integrase inhibitor; APT, activated prothrombin time; PTT, partial thromboplastin time; INR, international normal ratio. *p < 0.05.

### Increased platelet activation in HIV-infected individuals virologically suppressed through ART

**Figure 1:**
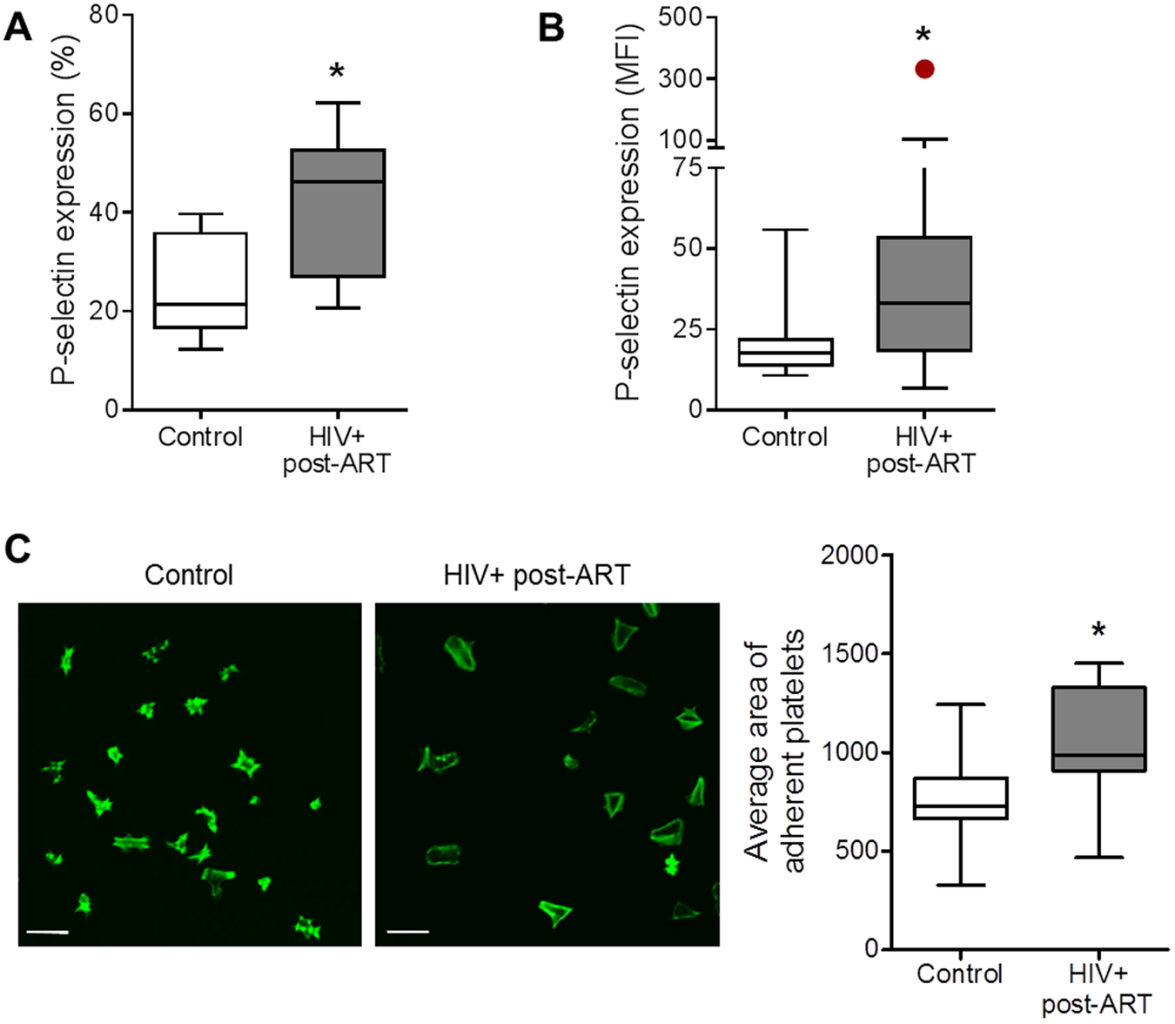
Increased platelet activation in HIV-infected individuals under stable cART. (**A**,**B**) The percentage of platelets with P-selectin surface expression (**A**) and the mean fluorescence intensity (MFI) for P-selectin labeling on platelets (**B**) were assessed in freshly-isolated platelets from healthy subjects (control) or from HIV-infected individuals under viral control (undetectable HIV-1 viral load in the peripheral blood). (**C**) Average area of spontaneous spreading on fibrinogen-coated surfaces by platelets from control or HIV-1-infected subjects labeled with phalloidin. Images are representative of eight HIV-infected individuals and healthy volunteers analyzed in parallel. Scale bars represent 10 µm. The horizontal lines on the box plots represent the median, the boxes limits represent the interquartile ranges and the whiskers indicate 5–95 percentile. The dot in panel B represents an outlier (p < 0.05 in Grubbs’ test) that was excluded from statistical analysis. The asterisk (*) signifies p < 0.05 compared to healthy volunteers.

To assess whether platelet activation was present in HIV-infected individuals virologically suppressed by ART, platelets from healthy volunteers and HIV-infected individuals were evaluated for P-selectin surface expression. The percent of P-selectin-positive platelets and the intensity of P-selectin labeling (mean fluorescence intensity, MFI) on platelets were significantly (p < 0.01) higher in samples from HIV-infected individuals [46.2% (IQR 27.0–52.7) and 33.0 MFI (IQR 18.3–53.4)] when compared to healthy volunteers [21.4% (IQR 16.7–35.8) and 17.7 MFI (IQR 13.9–22.9)] (Fig. 1A,B). Next, we evaluated the spreading area of platelets from HIV-infected individuals or healthy volunteers when adhered to fibrinogen-coated surfaces. For this assay, platelets were adhered on surfaces coated with high-density of fibrinogen (100 µg/mL) which leads to reduced platelet spreading at baseline^29^ allowing us to identify differential spreading between platelets from control or HIV-infected subjects. As shown in Fig. 1C, platelets from HIV-infected individuals under cART spread to a significantly (p < 0.05) increased area in response to fibrinogen when compared to platelets from healthy volunteers. Altogether, these data indicate increased platelet activation in HIV-1-infected subjects despite viral suppression through stable ART.

### Mitochondrial dysfunction and apoptosis in platelets from HIV-infected individuals under stable ART

**Figure 2:**
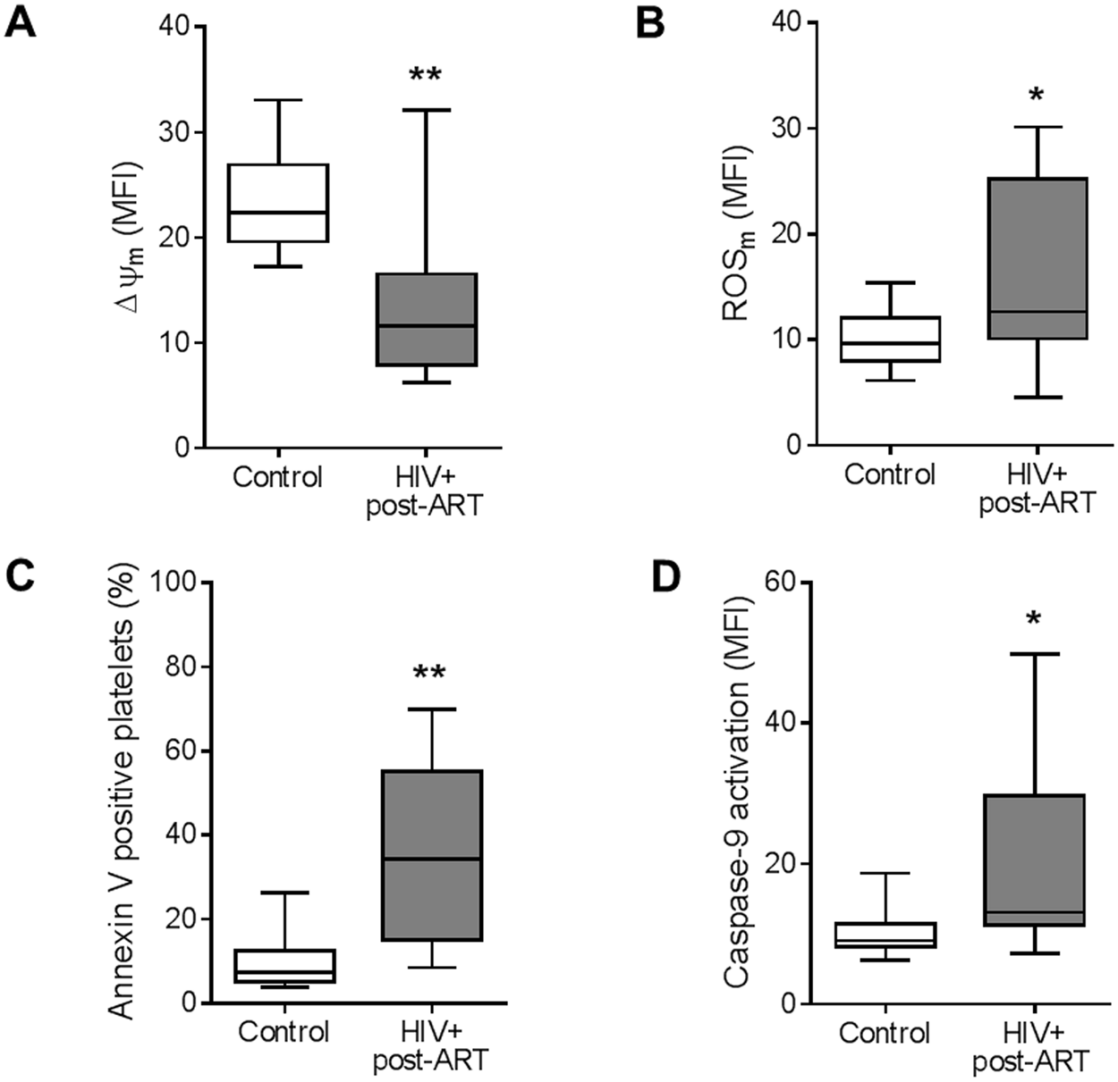
Mitochondrial dysfunction and apoptosis in platelets from HIV-infected individuals under stable cART. (**A**) The mean fluorescence intensity (MFI) for TMRE indicating the mitochondrial membrane potential (∆ψ_m_), (**B**) the MFI for MitoSoxRed indicating mitochondrial generation of reactive oxygen species (ROS_m_), (**C**) the percentage of platelets that bind annexin V, and (**D**) the MFI for the probe FLICA indicating caspase-9 activation were assessed in platelets freshly-isolated from healthy volunteers (control) or from HIV-infected individuals under viral control (HIV-1 viral load < 50 copies/ml in the peripheral blood). Box plots: The horizontal lines on the box plots represent the median, boxes limits indicate interquartile ranges and the whiskers indicate 5–95 percentile. The asterisk (*) signify p < 0.05 compared to healthy volunteers.

It has been previously demonstrated that increased platelet activation is associated to mitochondrial dysfunction and activation of the intrinsic pathways of apoptosis^30^. To evaluate the mitochondrial function in platelets from HIV-infected individuals, we used the mitochondrial targeted probes TMRE and MitoSox Red, which label mitochondrial membrane potential (ΔΨ_m_) and mitochondrial-derived ROS (ROS_m_), respectively. Mitochondrial specificity of the probes was confirmed by complete depolarization of platelet mitochondria with the proton ionophore FCCP (0.5 μM, 15 min) as previously reported^31^. As shown in Fig. 2A, basal ΔΨ_m_ was significantly (p < 0.01) reduced in platelets isolated from HIV-infected individuals [11.6 MFI (IQ 7.9–16.5)] when compared to healthy volunteers [22.4 MFI (IQ 19.6–26.9)]. Baseline production of ROS_m_ was also significantly (p < 0.05) higher in platelets from HIV-infected individuals than in controls [12.6 MFI (IQ 10.1–25.2) versus 9.6 MFI (IQ 7.9–12.1), respectively] (Fig. 2B).

Mitochondria are important regulators of the intrinsic pathway of apoptosis, and increased mitochondrial dysfunction strongly suggests that apoptotic pathways are more active in platelets^32^. To assess activation of apoptosis pathways, we evaluated phosphatidylserine exposure and caspase-9 activation in platelets from HIV-infected individuals and controls. Platelet phosphatidylserine exposure was significantly higher (p < 0.01) in HIV-infected individuals [34.3% (IQ 15.0–55.2)] compared to healthy volunteers [7.3% (IQ 5.2–12.4)] (Fig. 2C). In addition, caspase-9 activation was increased in platelets isolated from HIV-infected individuals [13.1 (IQ 11.2–29.8)] compared to healthy subjects [9.0 (IQ 8.2–11.5) (Fig. 2D). In association with collapsed mitochondrial function, these data indicate activation of the intrinsic pathway of apoptosis in platelets during HIV-1 infection, even with viral suppression by ART.

### Platelet exhaustion in HIV-infected individuals undergoing virological suppression through ART

**Figure 3:**
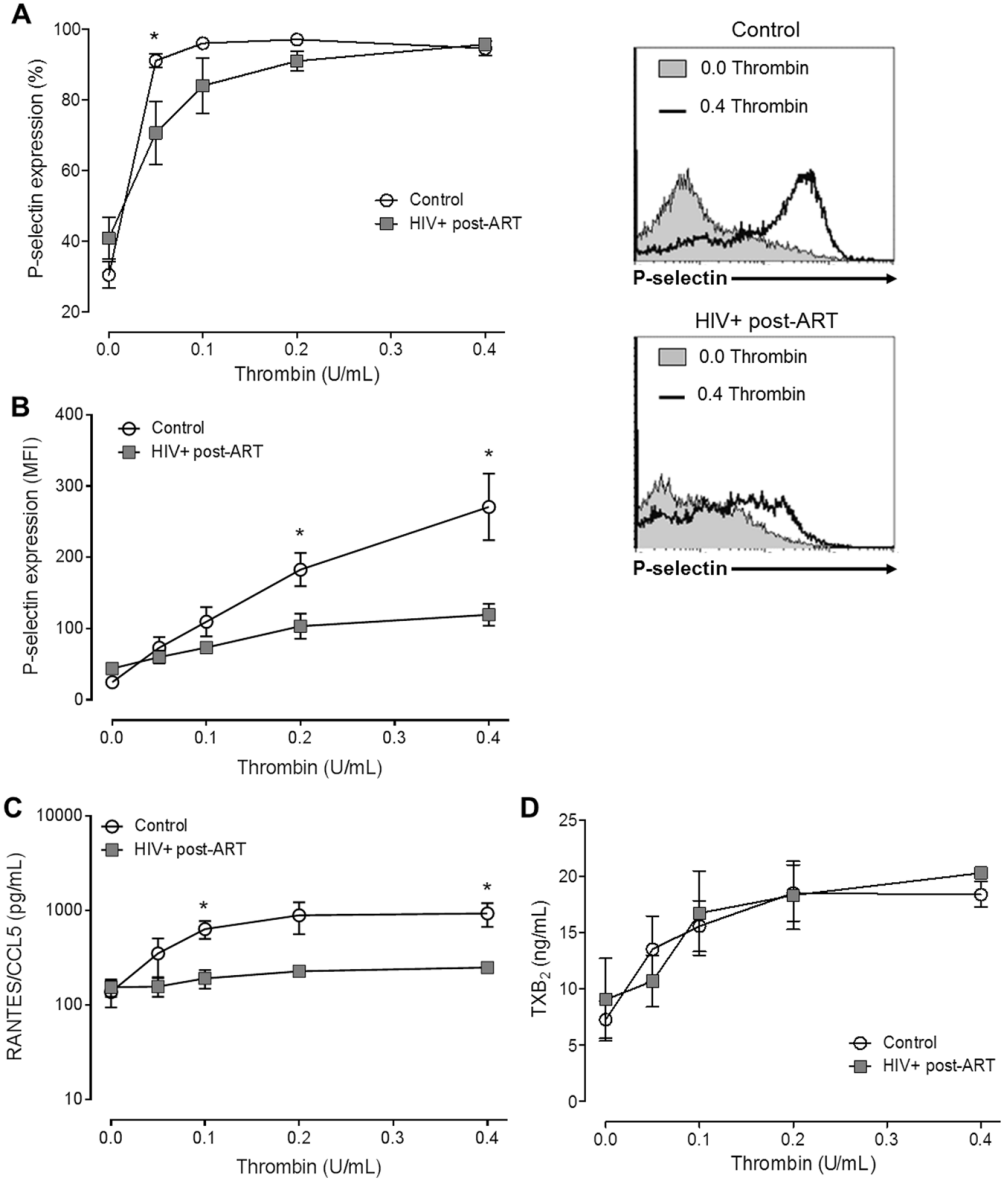
Platelet response to thrombin in platelets from HIV-infected individuals under stable cART and healthy volunteers. (**A**,**B**) The percentage of platelets with P-selectin surface expression (**A**) and the mean fluorescence intensity (MFI) for P-selectin labeling (**B**) on platelets from healthy subjects (control) or from HIV-infected individuals under viral control (HIV-1 viral load < 50 copies/ml in the peripheral blood) after stimulation with thrombin (0.05 U/mL, 0.1 U/mL, 0.2 U/mL and 0.4 U/mL). (**C,D**) The concentration of RANTES (**C**) or thromboxane B_2_ (**D**) in the supernatant of platelets from control or HIV-1-infected subjects stimulated with each concentration of thrombin. The dots indicate the mean ± standard error of the mean of 5–10 HIV-infected individuals or healthy volunteers. Representative histograms are shown. The asterisk (*) signifies p < 0.05 between platelets from control or healthy volunteers stimulated with the same concentration of thrombin.

Next, we determined the platelet response to thrombin stimulation in HIV-infected individuals under stable ART. Platelets from healthy donors or from HIV-infected individuals were incubated with 0.0 to 0.4 U/mL of thrombin according to described in material and methods. Even though platelets from HIV-infected individuals had increased P-selectin surface expression at baseline, P-selectin trafficking to surface in response to thrombin was lower in platelets from HIV-infected individuals compared to control subjects (Fig. 3A,B). Consistently, higher concentrations of thrombin were necessary to achieve 100% of P-selectin-expressing platelets in samples from HIV-infected individuals than in control (Fig. 3A). The intensity of P-selectin labeling on platelet surface (MFI) was significantly (p < 0.05) increased after thrombin stimuli in both healthy volunteers (270.9 ± 124.1 MFI versus 24.7 ± 11.3 MFI, for 0.4 U/ml of thrombin and unstimulated platelets, respectively) and HIV-infected individuals (119.6 ± 48.7 MFI versus 43.9 ± 23.8 MFI for 0.4 U/mL of thrombin and non-stimulated, respectively). However, P-selectin labeling on thrombin-stimulated platelets was significantly higher in platelets from healthy volunteers than HIV-infected individuals (Fig. 3B), even with 100% of platelets expressing P-selectin in both groups (Fig. 3A).

Thrombin stimuli also increased the secretion of the stored chemokine RANTES/CCL5 in platelets from both, HIV-infected individuals (248.9 ± 63.5 pg/ml versus 154 ± 71.3 pg/ml for 0.4 U/mL of thrombin and unstimulated platelets, respectively; p = 0.043) and healthy volunteers (930.4 ± 584.2 pg/ml versus 137.1 ± 95.4 pg/ml for 0.4 U/mL of thrombin and unstimulated platelets, respectively; p = 0.034). However, chemokine secretion was significantly (p < 0.05) lower in platelets derived from HIV-infected individuals compared to healthy volunteers (Fig. 3C). As both P-selectin and RANTES proteins are pre-formed and stored in platelet α-granules^16^, these data suggest exhaustion of platelet granules content in HIV-infected individuals under stable ART.

**Figure 4:**
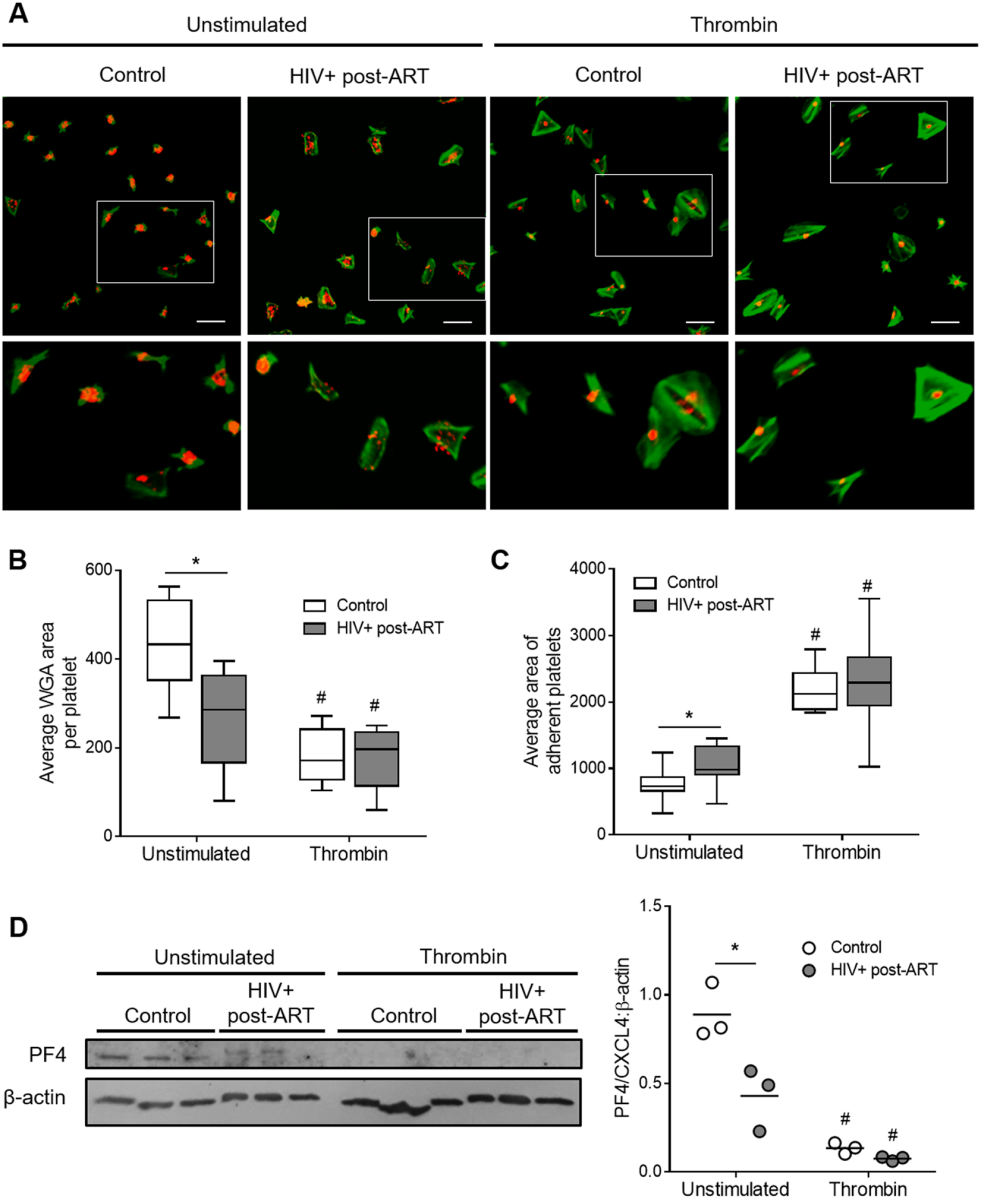
Thrombin-induced platelet spreading and degranulation in HIV-infected individuals under stable cART and healthy volunteers. (**A**) Platelets from healthy subjects (control) or HIV-infected individuals under viral control (HIV-1 viral load < 50 copies/ml in the peripheral blood) were stimulated with thrombin (0,0 or 0,4 U/mL), adhered to fibrinogen-coated surfaces and labeled with phalloidin (green) and wheat germ agglutinin (WGA – red) as described in methods. (**B**) Average area of WGA labeling per platelet number in each field for unstimulated or thrombin-stimulated platelets from control or HIV-1-infected subjects. (**C**) Average area of spontaneous or thrombin-induced platelet spreading on fibrinogen-coated surfaces in each condition. Images are representative of eight HIV-infected individuals and healthy volunteers analyzed in parallel. Scale bars represent 10 µm. The horizontal lines on the box plots represent the median, boxes limits indicate interquartile ranges and the whiskers indicate 5–95 percentile. (**D**) Western blot analysis for PF4/CXCL4 and β-actin in platelets from three healthy volunteers and three HIV-infected subjects under stable cART that were kept unstimulated or stimulated with 0.4 U/mL of thrombin. Dots represent the band intensity of PF4 corrected by β-actin expression in each experimental condition. Horizontal lines represent mean. The asterisk (*) signify p < 0.05 compared to healthy volunteers and # means p < 0.05 compared to unstimulated platelets.

To assess platelet exhaustion during HIV-1 infection in more depth, we labeled sialic acid residues in α-granules of fibrinogen-adhered platelets using wheat germ agglutinin (WGA) and quantified the area of labeled granules per platelet. As shown in Fig. 4A,B, platelets from HIV-infected individuals had reduced WGA stained area when compared to platelets from healthy volunteers. We then measured the granule-stored chemokine platelet factor 4 (PF4/CXCL4) through western blot, and observed lower PF4/CXCL4 quantities in platelets from HIV-infected subjects under stable cART compared to healthy volunteers (Fig. 4D). Thrombin stimulation significantly (p < 0.05) reduced WGA labeled area and PF4/CXCL4 expression to the same extent in both HIV and control platelets (Fig. 4A,B,D). Similarly, platelets from HIV-infected individuals increased their spreading area on fibrinogen after thrombin stimulation through the same extent than control platelets (Fig. 4A,C). We then measured the levels of thromboxane B_2_ (TXB_2_), a stable metabolite of the eicosanoid TXA_2_, synthesized after platelet stimulation with thrombin. As shown in Fig. 3D, platelet stimulation with 0.1 to 0.4 U/mL of thrombin significantly (p < 0.05) increased the synthesis of TXA_2_ compared to unstimulated platelets in both healthy volunteers and HIV-infected individuals. Taken together, these results indicate that platelets from HIV-infected individuals are responsive to thrombin, but secrete lower levels of granule stored factors (RANTES and P-selectin) because of exhausted granule content.

## Discussion

Despite viral suppression achieved through cART, HIV-infected individuals have a heightened risk for developing long-term complications, including cardiovascular and thrombotic events^3,7,11^. Emerging evidence suggests a role for persistent immune activation and dysfunction^10,14,33^, and platelet activation may play an important role in these outcomes. Here we show that HIV-infected individuals virally suppressed through cART had increased platelet activation and signs of apoptosis when compared to control subjects. Moreover, we observed lower P-selectin surface translocation and RANTES secretion, but not TXA_2_ synthesis, after thrombin stimulation of platelets from HIV-infected individuals compared to platelets from healthy volunteers. Chronic activation of platelets from HIV-infected individuals was associated with exhaustion of platelet granule content. Our data demonstrate that platelet activation and dysfunction persists in HIV-infected individuals undergoing viral suppression though cART.

Prior studies have demonstrated increased platelet activation in HIV-infected individuals^19–23,34^. Platelet surface P-selectin expression and plasma markers of platelet activation, such as soluble P-selectin, CD40-L and glycoprotein VI, are increased in HIV-infected individuals and decrease during the first weeks after cART initiation^19,35^. Here we show that, compared to healthy volunteers, platelet activation is still higher in HIV-infected individuals after at mean 60 months of ART.

Although HIV can direct activate platelets^36^, other mechanisms for platelet activation should be considered since, according to our data, platelet activation is also observed in virally suppressed HIV-infected individuals. One possible mechanism is microbial translocation, which is a central feature of HIV/AIDS pathogenesis and is still observed for years after antiretroviral treatment^37,38^. According to this hypothesis, platelets express functional TLR4^39^ and lipopolysaccharide binding to TLR4 on platelets triggers platelet activation^40^. Other possibilities are the increased generation of platelet activating factor (PAF) in HIV-1-infected subjects^41–43^ and/or the effects of antiretroviral treatment itself. Regarding the latter, abacavir-containing antiretroviral regimens have been associated with platelet hyperreactivity while raltegravir-based therapy was associated with reduced platelet activation^44–46^. Ritonavir-containing protease inhibitor-based cART has been also associated to increased platelet activation when compared to samples obtained before therapy initiation in a small cohort of protease-inhibitor naïve patients^47^. New studies are still necessary to better understand the effects of different cART regimens, in particular the new drugs on platelet responses.

Apoptosis of peripheral blood mononuclear cells from HIV-infected individuals occurs in parallel to collapse of ∆ψ_m_ and unbalanced ATP production^26,48^. Follow up of HIV-infected individuals initiating antiretroviral treatment shows significant improvement of mitochondrial function and leukocyte apoptosis, but this cell death phenotype persists under ART and an influence of the treatment itself has been considered^26,49,50^. We similarly observed mitochondrial dysfunction and apoptosis in platelets from HIV-infected individuals virologically suppressed by cART. Platelets from HIV-infected individuals showed decreased ∆ψ_m_, increased generation of ROS_m_, increased caspase-9 activity and increased phosphatydilserine exposure, strongly suggesting activation of the intrinsic pathway of apoptosis. Platelet mitochondrial dysfunction and apoptosis is related to thrombocytopenia in other diseases conditions as dengue and sepsis^31,51,52^. In the present study, we did not observe a relationship of platelet mitochondrial dysfunction and apoptosis with platelet counts (data not shown). Future studies with larger cohorts and greater numbers of thrombocytopenic HIV-infected individuals will be necessary to address the role of platelet apoptosis in HIV-1-associated thrombocytopenia.

We also observed that HIV-infected individuals under stable ART presented platelet exhaustion, with reduced P-selectin translocation and RANTES secretion, but normal TXA_2_ synthesis, in response to thrombin stimulation. Holme and colleagues^19^ have previously demonstrated lower secretion of RANTES by platelets from HIV-1-infected subjects after stimulation with thrombin receptor agonist peptide. In this study, RANTES secretion by platelets from HIV-infected individuals reached closer to normal levels after 12 weeks of antiretroviral treatment, even though remained different from healthy volunteers^19^. Accordingly, our results show lower RANTES secretion by thrombin-stimulated platelets from HIV-infected individuals even under stable ART. Similarly, reduced staining for platelet dense-granules has been recently reported in HIV-infected subjects compared to healthy volunteers^53^, suggesting that platelet dense-granules are also exhausted in HIV infection. In contrast to the lower platelet degranulation in response to thrombin, increased aggregation response due to ADP, collagen or arachidonic acid stimulation was reported in platelets from HIV-infected individuals under stable antiretroviral treatment^54^. These data suggest that persistent platelet activation in HIV-1-infected subjects virologically suppressed by ART may have as consequence not only a deficient release of granule-stored factors, but also hyperreactive aggregation response and thrombus formation.

Our study has limitations. Because of the cross-sectional design and small sample size in this study we could not address the potential effects of different ART regimens on platelet physiology. Also, we observed a small difference in age between HIV-infected individuals and controls. However, age is not classically associated with P-selectin expression in other studies^20,55^.

In summary, we report increased platelet activation, mitochondrial dysfunction and apoptosis in platelets from HIV-infected individuals under stable ART. We also provide evidence for platelet exhaustion as a possible consequence of chronic platelet activation during HIV-1 infection. To our knowledge, this is the first description of platelet apoptosis in HIV-1-infected subjects. These data demonstrate that platelets are persistently activated during HIV-1 infection, even though viral suppression is achieved through ART. Each of these cellular events potentially contributes to sustained inflammation and increased cardiovascular risk in HIV-infected individuals undergoing virological suppression though ART.

## Material and Methods

### Ethics Statement

The study was conducted in accordance with the Declaration of Helsinki. Written informed consent was obtained from all volunteers or their surrogate decision makers, prior to any study-related procedure. The study protocol was approved by the Institutional Review Board (Instituto Nacional de Infectologia Evandro Chagas – CAAE 0043.0.009.000-11; and University of Utah Molecular Medicine Program – IRB_00077138).

### Design and setting

Thirty six healthy volunteers and 30 HIV-infected individuals under stable ART were included from the outpatient clinic of the National Institute of Infectious Diseases Evandro Chagas (INI), Oswaldo Cruz Foundation (FIOCRUZ), Rio de Janeiro, Brazil or from the Molecular Medicine Program, University of Utah, Salt Lake City, Utah, USA. All samples were collected in the morning. Exclusion criteria for this study included age <18 years, current use of NSAID or other antithrombotic agents, history of myocardial infarction, co-infection with HCV, HBV or HTLV, co-existing cancer and co-existing active infection.

### Platelet Isolation

Platelets were isolated using the methods described by Hamburger & McEver^56^. Briefly, peripheral blood samples were drawn into acid-citrate-dextrose (ACD; 3 ml ACD/ 17 ml of blood) coated syringe. Samples were then centrifuged at 200 × g for 20 min to obtain platelet-rich plasma (PRP). PRP was centrifuged at 500 × g for 20 min in the presence of 100 nM PGE_1_ (Cayman Chemicals, Michigan, USA). The supernatant was discarded and platelet pellet was resuspended in 25 mL of PIPES/saline/glucose (5 mM piperazine*-*N,N*-*bis[2-ethanesulfonic acid], 145 mM NaCl, 4 mM KCl, 50 mM Na_2_HPO_4_, 1 mM MgCl_2_−6 H_2_O, and 5.5 mM glucose) containing 100 nM of PGE_1_. The platelet suspension was recentrifuged at 500 × g for 20 min, the supernatant was discarded, and the pellet was re-suspended in medium 199 (M199, Lonza Biologics; Basel, Switzerland). All centrifugations were performed in the absence of a brake. The purity of the platelet preparations (greater than 99% CD41^+^ events) was confirmed by flow cytometry.

### Flow Cytometry Analysis

Freshly isolated platelets (1 × 10^7^) were re-suspended in 500 µL of modified Tyrode’s (137 mMNaCl, 2.68 mM KCI, 5 mM HEPES, 1 mM MgCl_2_, 11.9 mM NaHCO_3_, 0.42 mM NaH_2_PO_4_ e 4.7 mM glucose; pH 7.4) and analyzed through flow cytometry. The following indices were assessed: P-selectin (CD62-P) surface expression was determined by incubating (37 °C for 30 min) platelets with a PE- or FITC-conjugated antibody (1:25 µg/mL) that targets CD62P (BD Pharmingen, San Diego, CA, USA); mitochondrial membrane potential (ΔΨ_m_) was measured using the cationic probe Tetramethylrhodamine ethyl ester (TMRE; Fluka Analytical, St. Gallen, Switzerland) (100 nM at 37 °C for 10 min); mitochondria-generated reactive oxygen species (ROS_m_) were detected using the cationic probe MitoSOX Red (Invitrogen, Carlsbad, CA, USA) (2.5 µM at 37 °C for 10 min); activation of caspase-9 was assessed using the fluorescent probe green FAM-LEDH-FMK, FLICA according to the manufacturer’s instructions (Immunochemistry Technologies, Bloomington, MN USA.) and; phosphatidylserine exposure on the surface of platelets was determined using FITC-conjugated Annexin V according to the manufacturer’s instructions (BD Pharmingen). Platelets were gated by specific binding to CD41 and characteristic forward and side scattering. A minimum of 10,000 gated events were acquired using a FACS Calibur flow cytometer (BD Bioscience). For each condition, we defined the appropriate color compensations and determined the mean fluorescence intensity (MFI) or the percentage of positive platelets.

### *In vitro* platelet stimulation

Platelets (05 × 10^6^ in 100 µL) from healthy uninfected donors or from HIV-infected individuals were stimulated with 0.05, 0.1, 0.2 or 0.4 U/mL of thrombin (Sigma, T1063; MO, USA), or kept in medium only. Platelet P-selectin surface expression and the concentration of RANTES/CCL5 and TXB_2_ in the supernatants were evaluated 1 hour after thrombin stimulation.

### Quantification of RANTES and TXB2 in supernatant

The content of the chemokine RANTES/CCL5 and the eicosanoid TXB_2_ were measured in the supernatants from thrombin-stimulated platelets using commercially available ELISA and EIA Kits, respectively, according to the manufacturer’s instructions (R&D systems and Cayman chemicals, respectively).

### Static platelet adhesion assay and confocal microscopy analysis

For platelet spreading experiments, borosilicate glass Lab-Tek chambers were coated with fibrinogen (100 µg/ml, 1 h at 37 °C). Platelets (1 × 10^7^) from HIV-infected individuals or healthy volunteers were stimulated with thrombin (0,0 or 0,4 U/mL) for 20 min prior to exposure to fibrinogen coated surfaces. After 1 hour incubation at 37 °C, platelets were fixed in 4% paraformaldehyde and washed three times with PBS. Platelets were stained with WGA (Wheat Germ Agglutinin, Alexa Fluor™ 555 #W32464) and phalloidin (Alexa Fluor™ 488 Phalloidin #A12379) for 30 min at room temperature and imaged using FV1000 1 × 81 confocal microscope and FluoView software (Olympus). Images were analyzed using ImageJ version 1.49 m software (National Institutes of Health, USA) with Java 1.6.0_24 (64-bit). We developed a macro to analyze the total WGA stained area (red) in each field adjusting the same parameters of color balance, contrast, background and noise. Images were processed so that the threshold setting for quantifying the total and relative area of WGA staining excluded most of interferences from the image acquisition. A different macro was developed for the counting of platelet numbers in each field (phalloidin – green). In this case, threshold was adjusted to consider the differential platelet spreading among groups. Then, total WGA stained area was normalized by the number of platelets in each field and the mean of these measurements was plotted for HIV-infected individuals or healthy volunteers.

### Western blot

Freshly isolated platelets from HIV-infected subjects or healthy volunteers were kept unstimulated or stimulated with 0.4 U/mL of thrombim for 1 h. Platelets were them lysed (0.15MNaCl, 10 mM Tris pH 8.0, 0.1 mM EDTA, 10% Glicerol and 0.5% triton X-100) in the presence of protease inhibitors (Roche, Indianapolis, IN). Platelet proteins

(17.5 μg) were separated by 15% sodium dodecyl sulfate-polyacrylamide gel electrophoresis (SDS-PAGE) and transferred into nitrocellulose membrane. The membrane was blocked in Tris-buffered saline (TBS) supplemented with 0.1% Tween 20 (TBS-T) and 5% low-fat dried milk for 1 h before incubation overnight with mouse anti-human PF4/CXCL4 (R&D Systems) (1:1000) or for 1 h with mouse anti-human β-actin (Sigma Aldrich) (1:10,000) primary antibodies. After washing five times in TBS-T, the membrane was revealed using peroxidase-conjugated secondary antibodies against mouse immunoglobulin (Vector) (1:10,000, 1 h).

### Statistical Analysis

All statistical analyses were performed using GraphPad Prism 7.0 software (San Diego, CA). Kolmogorov-Smirnov or Shapiro-Wilk normality test were used to determine whether samples followed normal distribution. T test was used for comparison between two experimental groups that followed a parametric distribution. Mann Whitney U test was used to determine whether differences were present between two experimental groups that followed a nonparametric distribution. A paired two-tail t-test was used to compare thrombin-stimulated with non-stimulated platelets from HIV-infected individuals or control group. Correlations were assessed using the Pearson’s test.

## References

[1] Lewden C (2007). HIV-infected adults with a CD4 cell count greater than 500 cells/mm3 on long-term combination antiretroviral therapy reach same mortality rates as the general population. J Acquir Immune Defic Syndr.

[2] Dore GJ (2002). Impact of highly active antiretroviral therapy on individual AIDS-defining illness incidence and survival in Australia. J Acquir Immune Defic Syndr.

[3] Marin B (2009). Non-AIDS-defining deaths and immunodeficiency in the era of combination antiretroviral therapy. AIDS.

[4] Hasse B (2011). Morbidity and aging in HIV-infected persons: the Swiss HIV cohort study. Clin Infect Dis.

[5] Casper C (2011). The increasing burden of HIV-associated malignancies in resource-limited regions. Annu Rev Med.

[6] Grulich AE, van Leeuwen MT, Falster MO, Vajdic CM (2007). Incidence of cancers in people with HIV/AIDS compared with immunosuppressed transplant recipients: a meta-analysis. Lancet.

[7] Goehringer F (2017). Causes of Death in HIV-Infected Individuals with Immunovirologic Success in a National Prospective Survey. AIDS Res Hum Retroviruses.

[8] d’Arminio A (2004). Cardio- and cerebrovascular events in HIV-infected persons. AIDS.

[9] Lang S (2012). HIV replication and immune status are independent predictors of the risk of myocardial infarction in HIV-infected individuals. Clin Infect Dis.

[10] Lichtenstein KA (2010). Low CD4 + T cell count is a risk factor for cardiovascular disease events in the HIV outpatient study. Clin Infect Dis.

[11] Lazar, R. *et al*., Hospitalization rates among people with HIV/AIDS in New York City, 2013. *Clin Infect Dis* (2017). 10.1093/cid/cix343 28444155

[12] Kuller LH (2008). Inflammatory and coagulation biomarkers and mortality in patients with HIV infection. PLoS Med.

[13] McDonald B (2013). Persistently elevated serum interleukin-6 predicts mortality among adults receiving combination antiretroviral therapy in Botswana: results from a clinical trial. AIDS Res Hum Retroviruses.

[14] Tien PC (2010). Inflammation and mortality in HIV-infected adults: analysis of the FRAM study cohort. J Acquir Immune Defic Syndr.

[15] Semple JW, Italiano JE, Freedman J (2011). Platelets and the immune continuum. Nat Rev Immunol.

[16] Vieira-de-Abreu A, Campbell RA, Weyrich AS, Zimmerman GA (2012). Platelets: versatile effector cells in hemostasis, inflammation, and the immune continuum. Semin Immunopathol.

[17] Weyrich AS (1995). Monocyte tethering by P-selectin regulates monocyte chemotactic protein-1 and tumor necrosis factor-alpha secretion. Signal integration and NF-kappa B translocation. J Clin Invest.

[18] Hottz ED (2014). Platelet activation and apoptosis modulate monocyte inflammatory responses in dengue. J Immunol.

[19] Holme PA (1998). Enhanced activation of platelets with abnormal release of RANTES in human immunodeficiency virus type 1 infection. FASEB J.

[20] Mayne E (2012). Increased platelet and microparticle activation in HIV infection: upregulation of P-selectin and tissue factor expression. J Acquir Immune Defic Syndr.

[21] Nkambule, B. B., Davison, G. and Ipp, H., The value of flow cytometry in the measurement of platelet activation and aggregation in human immunodeficiency virus infection. *Platelets*, 1 (2014). 10.3109/09537104.2014.909021 24831969

[22] Nkambule BB, Davison GM, Ipp H (2015). The evaluation of platelet function in HIV infected, asymptomatic treatment-naive individuals using flow cytometry. Thromb Res.

[23] Trevillyan JM (2016). Decreased levels of platelet-derived soluble glycoprotein VI detected prior to the first diagnosis of coronary artery disease in HIV-positive individuals. Platelets.

[24] de Oliveira Pinto LM (2002). Increased sensitivity of T lymphocytes to tumor necrosis factor receptor 1 (TNFR1)- and TNFR2-mediated apoptosis in HIV infection: relation to expression of Bcl-2 and active caspase-8 and caspase-3. Blood.

[25] Fraietta JA (2013). Type I interferon upregulates Bak and contributes to T cell loss during human immunodeficiency virus (HIV) infection. PLoS Pathog.

[26] Bociaga-Jasik M (2013). Mitochondrial function and apoptosis of peripheral mononuclear cells (PBMCs) in the HIV infected patients. Curr HIV Res.

[27] Leytin V (2006). Thrombin-triggered platelet apoptosis. J Thromb Haemost.

[28] Mason KD (2007). Programmed anuclear cell death delimits platelet life span. Cell.

[29] Jirouskova M, Jaiswal JK, Coller BS (2007). Ligand density dramatically affects integrin alpha IIb beta 3-mediated platelet signaling and spreading. Blood.

[30] Jobe SM (2008). Critical role for the mitochondrial permeability transition pore and cyclophilin D in platelet activation and thrombosis. Blood.

[31] Hottz ED (2013). Dengue induces platelet activation, mitochondrial dysfunction and cell death through mechanisms that involve DC-SIGN and caspases. J Thromb Haemost.

[32] Estaquier J, Vallette F, Vayssiere JL, Mignotte B (2012). The mitochondrial pathways of apoptosis. Adv Exp Med Biol.

[33] van Lelyveld SF (2012). Long-term complications in patients with poor immunological recovery despite virological successful HAART in Dutch ATHENA cohort. AIDS.

[34] Hottz ED, Bozza FA, Bozza PT (2018). Platelets in Immune Response to Virus and Immunopathology of Viral Infections. Front Med (Lausanne).

[35] O’Halloran JA (2015). The effect of initiation of antiretroviral therapy on monocyte, endothelial and platelet function in HIV-1 infection. HIV Med.

[36] Chaipan C (2006). DC-SIGN and CLEC-2 mediate human immunodeficiency virus type 1 capture by platelets. J Virol.

[37] Brenchley JM (2006). Microbial translocation is a cause of systemic immune activation in chronic HIV infection. Nat Med.

[38] Rajasuriar R (2010). Biological determinants of immune reconstitution in HIV-infected patients receiving antiretroviral therapy: the role of interleukin 7 and interleukin 7 receptor alpha and microbial translocation. J Infect Dis.

[39] Andonegui G (2005). Platelets express functional Toll-like receptor-4. Blood.

[40] Shashkin PN (2008). Lipopolysaccharide is a direct agonist for platelet RNA splicing. J Immunol.

[41] Tsoupras AB (2012). Platelet-activating factor and its basic metabolic enzymes in blood of naive HIV-infected patients. Angiology.

[42] Papakonstantinou VD (2014). *In vivo* effect of two first-line ART regimens on inflammatory mediators in male HIV patients. Lipids Health Dis.

[43] Kelesidis T (2015). The Role of Platelet-Activating Factor in Chronic Inflammation, Immune Activation, and Comorbidities Associated with HIV Infection. AIDS Rev.

[44] Satchell CS (2011). Increased platelet reactivity in HIV-1-infected patients receiving abacavir-containing antiretroviral therapy. J Infect Dis.

[45] Falcinelli E (2013). *In vivo* platelet activation and platelet hyperreactivity in abacavir-treated HIV-infected patients. Thromb Haemost.

[46] Tunjungputri RN (2014). Reduced platelet hyperreactivity and platelet-monocyte aggregation in HIV-infected individuals receiving a raltegravir-based regimen. AIDS.

[47] von Hentig N (2008). Platelet-leucocyte adhesion markers before and after the initiation of antiretroviral therapy with HIV protease inhibitors. J Antimicrob Chemother.

[48] Sternfeld T, Tischleder A, Schuster M, Bogner JR (2009). Mitochondrial membrane potential and apoptosis of blood mononuclear cells in untreated HIV-1 infected patients. HIV Med.

[49] de Oliveira Pinto LM (2002). Lack of control of T cell apoptosis under HAART. Influence of therapy regimen *in vivo* and *in vitro*. AIDS.

[50] Piconi S (2010). Immune activation, apoptosis, and Treg activity are associated with persistently reduced CD4 + T-cell counts during antiretroviral therapy. AIDS.

[51] Alonzo MT (2012). Platelet apoptosis and apoptotic platelet clearance by macrophages in secondary dengue virus infections. J Infect Dis.

[52] Grundler K (2014). Platelet mitochondrial membrane depolarization reflects disease severity in patients with sepsis and correlates with clinical outcome. Crit Care.

[53] Marcantoni E (2018). Platelet Transcriptome Profiling in HIV and ATP-Binding Cassette Subfamily C Member 4 (ABCC4) as a Mediator of Platelet Activity. JACC Basic Transl Sci.

[54] O’Brien M (2013). Aspirin attenuates platelet activation and immune activation in HIV-1-infected subjects on antiretroviral therapy: a pilot study. J Acquir Immune Defic Syndr.

[55] Choudhury A (2008). Soluble CD40 ligand, platelet surface CD40 ligand, and total platelet CD40 ligand in atrial fibrillation: relationship to soluble P-selectin, stroke risk factors, and risk factor intervention. Chest.

[56] Hamburger SA, McEver RP (1990). GMP-140 mediates adhesion of stimulated platelets to neutrophils. Blood.

